# Overweight and Obesity in Local Media: An Analysis of Media Coverage in CDC-Funded Communities

**DOI:** 10.5888/pcd14.170107

**Published:** 2017-12-07

**Authors:** Christopher N. Thomas, Derek Inokuchi, Thomas Lehman, Rebecca Ledsky, Andre Weldy

**Affiliations:** 1 Centers for Disease Control and Prevention, National Center for Chronic Disease Prevention and Health Promotion, Division of Nutrition, Physical Activity, and Obesity, Atlanta, Georgia; 2 FHI 360 Social Marketing and Communication, Washington, District of Columbia

## Abstract

We conducted a content analysis of newspaper and television news coverage in Centers for Disease Control and Prevention (CDC) grantee locations from June 2011 through May 2013. After searching 2 databases for news stories related to overweight or obesity, we coded and analyzed stories for valence (how the author/reporter framed overweight and obesity control strategies), descriptors, causes and solutions, and populations mentioned. Of almost 3,000 stories analyzed, most had a neutral or positive valence, depicted overweight and obesity as epidemic, discussed individual causes and environmental solutions most frequently, and mentioned children most often. Earned media can be part of addressing overweight and obesity by emphasizing prevention and by emphasizing both environmental and individual causes and solutions.

## Objective

Obesity may be caused by multiple factors (1). From 2011 through 2014, the Centers for Disease Control and Prevention (CDC) provided community-level funding to prevent chronic diseases and reduce obesity by using strategies focused on physical activity and nutrition (2,3). Most Americans follow local news through television and print newspaper (4), and news items make public health issues visible (5). CDC grantees used earned media strategies (eg, press releases, contacting reporters) to inform and obtain support for overweight and obesity program efforts. We wanted to understand the volume of news items related to overweight and obesity and how overweight and obesity were discussed in local news, including story valence (how the author/reporter framed overweight and obesity control strategies), descriptors, causes and solutions, and populations mentioned.

## Methods

We conducted a content analysis of newspaper and television news items for CDC grantees funded by Community Transformation Grants (2011–2014; n = 101) and Racial and Ethnic Approaches to Community Health Demonstration Projects (2012–2014; n = 2) (no communities received funding for both) (2,3).

We identified 316 print-only, English language (US-based) newspapers from the Nexis database (6) and identified television sources for 134 designated media markets from the News Data Services database (7). Nine grantees had no media outlets in the newspaper search, and 2 had none in the television search. Radio transcript data were not available. We excluded national media outlets and online-only newspapers and magazines because of our focus on local news.

We searched for newspaper stories and transcripts of television stories longer than 30 seconds from June 2011 through May 2013 to capture grantees’ initial implementation activities. Search strings included any elements of “overweight,” “obese,” “obesity,” and combinations of these (eg, overweight or obes*). We omitted transcripts for television coverage shorter than 30 seconds to avoid previews.

We coded all newspaper items found (n = 1,831; mean, 76 news items per month). For television transcripts (n = 49,912), we coded simple random samples (n = 2,513; mean, 105 news items per month; 5% per month) because of the volume found. All items (n = 4,344) were coded for 1) date and time, 2) location, 3) media outlet name, and 4) whether an item was “primarily about” overweight or obesity, “mentioned” overweight or obesity, or was off-topic. News items primarily about overweight or obesity were further coded for 1) valence; 2) descriptions; 3) discussion of causes and solutions; and 4) populations mentioned (Table).

**Table:** Coding Criteria^a^ for Newspaper and Television Coverage Primarily Related to Overweight or Obesity, Analysis of Media Coverage in CDC-Funded Communities, 2011–2013

Criteria	Definition or Examples
Valence^b^	Positive: reinforces CDC-aligned health message; expresses clear viewpoint supporting message Neutral: statement of facts, no particular positive or negative tone Negative: counters CDC-aligned health message, expresses clear negative viewpoint Mixed: presents opposing views within the same article
Overweight and obesity descriptors	Epidemic (eg, heightened significance; obesity is a national crisis) Preventable (eg, obesity can be prevented) Not a problem (eg, obesity prevalence is exaggerated)
**Causes of overweight and obesity**	
Individual	Sedentary lifestyle, physically inactive, or high amount of screen time (eg, television-watching, computer use) Lack of motivation Consuming unhealthy food or drink or too much food or drink
Environmental	Fewer school physical education classes Community design, urban planning (eg, no sidewalks, transportation, parks) Cost barriers (eg, gym membership, fitness equipment, price of healthy food) Limited healthy choices in stores, workplace, schools, restaurants, food ingredients (eg, *trans* fats) Large portion sizes
**Solutions to overweight and obesity **	
Individual	Increasing physical activity (eg, at least 30 min/d) Reducing screen time (eg, TV-watching, computer use) Making individual dietary changes (eg, eat more low fat foods; eat smaller portions, vegetables, breakfast) Decrease consumption of unhealthy beverages
Environmental	Community design and urban planning (eg, safe streets, sidewalks, bike paths, parks) Supporting school physical education classes or workplace physical activity opportunities Providing education on physical activity or nutrition Addressing cost-related barriers to physical activity Reducing food portion sizes Increasing healthy food choices or ingredients Providing more access to healthy foods Address cost-related barriers to healthy food consumption
**Population focus**	
Populations mentioned	Children and adolescents Blacks or African Americans Hispanics or Latinos Asians American Indians or Alaska Natives Low-income or below poverty level

Abbreviation: CDC, Centers for Disease Control and Prevention.

a Additional coding criteria, definitions, and examples used for television and print newspaper items initially coded as “primarily about” overweight and obesity.

b Refers to how an author/reporter framed overweight and obesity control strategies in the newspaper article or television transcript.

We achieved 90% intercoder agreement with a sentinel coder and with all coders before coding started. Spot checks were performed during coding. We generated descriptive statistics using SAS, version 9.3 (SAS Institute, Inc). We used χ^2 ^tests to determine differences in how overweight and obesity were discussed, by media type. Results were considered significant at *P* < .01.

## Results

Of the 4,344 news items from June 2011 through May 2013 that we reviewed, 2,978 (69%) were found to be primarily about overweight or obesity (1,139 newspaper articles, 1,839 television transcripts). Most items coded (95% of newspaper, 98% of television) had a neutral or positive valence for overweight or obesity control strategies. More newspaper items were written from a positive valence (24% positive, 71% neutral, 1% negative, 4% mixed) than were television items (1% positive, 97% neutral, 0% negative, 2% mixed) (*P* < .001). More newspaper items (43%) described overweight or obesity as an epidemic than television items did (13%) (*P* < .001). Few items described overweight or obesity as being preventable (newspaper, 10%; television, 10%) or as an exaggerated issue (range: <1%–2%).

Individual causes of overweight or obesity (eg, overeating) were mentioned more frequently than environmental causes (eg, lack of physical education classes in schools) (newspaper: 51% vs 32%, *P* < .001; television: 20% vs 6%, *P* < .001) (Figure). However, environmental solutions (eg, increased availability of healthy food options) were discussed more often than individual solutions (eg, eat smaller portions) (newspaper: 75% vs 67%, *P* < .001; television: 29% vs 26%, *P* < .006). More newspaper items than television items mentioned an individual cause (*P* < .001), an environmental cause (*P* < .001), or both (*P* < .001). Similarly, more newspaper items than television items mentioned an individual solution (*P* < .001), an environmental solution (*P* < .001), or both (*P* < .001).

**Figure:**
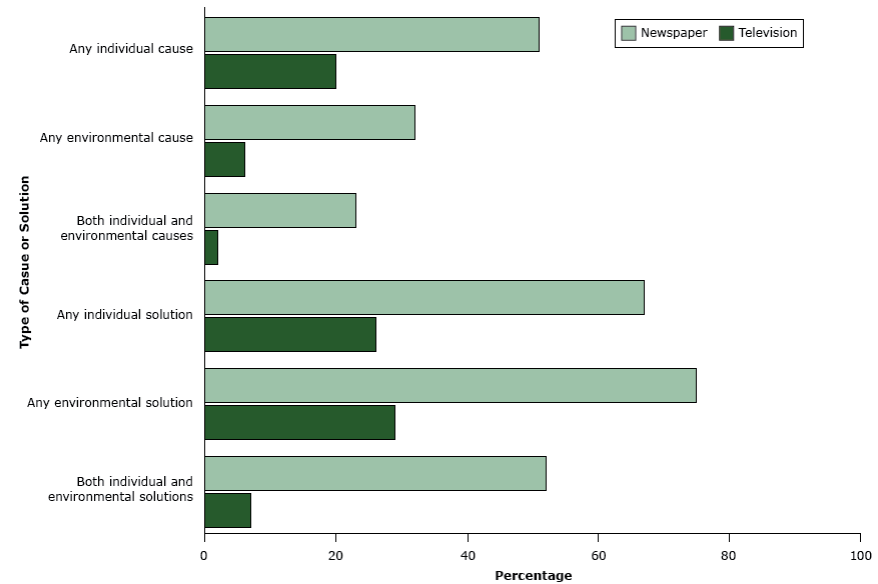
Causes of and solutions for overweight or obesity mentioned in newspaper and television news items, June 2011 through May 2013. The Nexis database (6) and News Data Services (7) database were searched for print newspaper articles and for transcripts of television coverage longer than 30 seconds. News items were coded and analyzed for mention of overweight or obesity causes or solutions. **Table:** 

Cause or Solution	Newspaper, %	Television, %
Any individual cause	51	20
Any environmental cause	32	6
Both individual and environmental causes	23	2
Any individual solution	67	26
Any environmental solution	75	29
Both individual and environmental solutions	52	7

Children were the most mentioned population (newspaper: 70%, television 50%; *P* < .001). The most frequently mentioned racial/ethnic populations were Hispanic or Latino (newspaper: 9%, television: 1%) and African American (newspaper: 8%, television: 1%).

## Discussion

Americans continue to rely on local news outlets to stay informed. The Pew Research Center reported that in 2016, 7 in 10 Americans followed local news and more than 80% had confidence in local news organizations (4). Our results show that local newspaper and television stories from June 2011 through May 2013 had a mostly neutral or positive valence. This finding may suggest that local earned media outlets are interested in topics related to public health and to overweight and obesity. Because local journalists rely on suggestions from a source and from press conferences and press releases (8), health agencies can share news releases with community-specific data or information (9).

We also found that overweight and obesity were described more often as an epidemic, less often as preventable, and with more discussion of individual causes and environmental solutions. An experimental study using vignettes about obesity’s causes found support for individual causes of obesity, whereas environmental causes were viewed as inaccurate and biased (10). As a result, the public may be exposed to more messages about individual causes for overweight and obesity. To address this, health agencies can create messages that emphasize that overweight and obesity can be prevented and messages that include both individual and environmental causes and solutions. This approach may build on past news coverage and thereby transition the overweight and obesity discussion from an epidemic to a preventable disease with both individual and environmental causes and solutions (eg, Tier 2, Health Impact Pyramid — Changing the Context to Encourage Healthy Decisions) (11).

The findings described here are from local news media from CDC-funded, community-level efforts focused on overweight and obesity. Our approach was unique by examining news stories related to overweight and obesity for multiple local media channels (ie, newspaper and television) in multiple locations across the United States. Future research could explore 1) whether public perception or news coverage changed regarding overweight and obesity causes and solutions and 2) the volume and impact of grantee efforts to obtain earned media coverage in relation to the coverage received.
